# Benchmarking different approaches for Norovirus genome assembly in metagenome samples

**DOI:** 10.1186/s12864-021-08067-2

**Published:** 2021-11-24

**Authors:** Azahara Fuentes-Trillo, Carolina Monzó, Iris Manzano, Cristina Santiso-Bellón, Juliana da Silva Ribeiro de Andrade, Roberto Gozalbo-Rovira, Ana-Bárbara García-García, Jesús Rodríguez-Díaz, Felipe Javier Chaves

**Affiliations:** 1 Unit of Genomics and Diabetes. Research Foundation of Valencia University Clinical Hospital- INCLIVA, Valencia, Spain; 2 Department of Microbiology, School of Medicine, University of Valencia, Valencia, Spain; 3 Laboratory of Comparative and Environmental Virology, Oswaldo Cruz Institute, Rio de Janeiro, Brazil; 4 Spanish Biomedical Research Network in Diabetes and Associated Metabolic Disorders (CIBERDEM), Madrid, Spain; 5 Sequencing Multiplex S.L., Valencia, Spain

**Keywords:** Norovirus, Genome de-novo assembly, Metagenomics

## Abstract

**Background:**

Genome assembly of viruses with high mutation rates, such as Norovirus and other RNA viruses, or from metagenome samples, poses a challenge for the scientific community due to the coexistence of several viral quasispecies and strains. Furthermore, there is no standard method for obtaining whole-genome sequences in non-related patients. After polyA RNA isolation and sequencing in eight patients with acute gastroenteritis, we evaluated two de Bruijn graph assemblers (SPAdes and MEGAHIT), combined with four different and common pre-assembly strategies, and compared those yielding whole genome Norovirus contigs.

**Results:**

Reference-genome guided strategies with both host and target virus did not present any advantages compared to the assembly of non-filtered data in the case of SPAdes, and in the case of MEGAHIT, only host genome filtering presented improvements. MEGAHIT performed better than SPAdes in most samples, reaching complete genome sequences in most of them for all the strategies employed. Read binning with CD-HIT improved assembly when paired with different analysis strategies, and more notably in the case of SPAdes.

**Conclusions:**

Not all metagenome assemblies are equal and the choice in the workflow depends on the species studied and the prior steps to analysis. We may need different approaches even for samples treated equally due to the presence of high intra host variability. We tested and compared different workflows for the accurate assembly of Norovirus genomes and established their assembly capacities for this purpose.

**Supplementary Information:**

The online version contains supplementary material available at 10.1186/s12864-021-08067-2.

## Background

Many viruses have high mutation and recombination rates, producing heterogeneous mixtures of viral strains. This rapid evolution favors the development of functional advantages such as evasion of the host immune response [1–4] and vaccine/drug resistance [5, 6]. Characteristics of infection and pathogenicity in these viruses are also influenced by selective pressure [7–9].

The heterogeneity underlying viral genomes complicates their characterization using sequencing experiments. Over recent decades, high-throughput sequencing (HTS) has emerged as an important resource for viral studies [10–12].

Among viruses with high mutation rates and presence of large numbers of genetic variations or quasispecies are Noroviruses, a group of the Caliciviridae family. They are positive and single-stranded RNA viruses without a lipid envelope, with genome lengths varying from 7.5 to 7.7 Kb [13]. These viruses are known to be highly pathogenic and infectious, and are responsible for most cases of acute gastroenteritis (50 % of all outbreaks worldwide), although symptoms are generally non-lethal [14]. Their genome is composed of three open reading frames (ORF), the first one encoding a polyprotein cleaved into six non-structural proteins including an RNA-dependent RNA polymerase (RdRp) [15]. ORF1 overlaps in a short region with ORF2, which encodes for the capsid protein VP1 (major capsid protein) while ORF3 encodes for VP2 (minor capsid protein) [16]. Of the ten currently identified genogroups, in humans the represented genogroups causing infections are GI, GII, GIV, and recently, GVIII and GIX [17]. These genogroups are established based on a minimum 43 % difference between VP1-coding sequences [17, 18]. However, 60 % of norovirus infections are attributable to genotype GII.4 [16, 19, 20]. As with other RNA viruses, the intra-genus variability of Noroviruses provides them with fast evolving capacity and makes their characterization and sequencing challenging [21–23].

Nowadays, HTS-based short read assemblers are widely used for reconstructing bacterial and viral genomes. Although HTS platforms and bioinformatics methods have evolved over the past few years, “de novo” assembly is still arduous and computationally expensive. Many of these assemblers are based on de Bruijn graph methods [24]. The strategy is to generate substrings of length k (k-mers) and form a path with overlapping sequences, constituting a graph and thus generating large contigs to reconstruct genomic regions [24, 25]. These computational algorithms were designed with the increasing use of short-read sequencing approaches to obtain contigs for assembly into scaffolds. Gene-centric and genome-centric assemblies require different approaches, as there are different ways to tackle short-read sequencing in metagenomics. SPAdes and MEGAHIT are frequently used de Bruijn graph-based assemblers in genome-centric studies due to their large contig yield [26].

One notable difference between SPAdes and MEGAHIT is that the former is more computationally expensive, working with the whole set of sequences in all assembly iterations, whereas the latter saves computational resources by considering only k-mers occurring over a determined cutoff length. metaSPAdes (SPAdes with --meta flag) is an alternative since it constructs a consensus sequence from different strain variants [27].

Compared with other organisms, virus genomes are generally difficult to assemble, not only because of the interspecies variability present in metagenome samples, but also due to the high genetic variability presented in viral particles [28–32]. Our goal was to reconstruct the genomes of Norovirus strains from stool samples from patients with gastroenteritis and diarrhea, testing different workflows and evaluating the use of read binning along with metagenome assembly. Our aim was to obtain large contigs spanning the whole genome of Norovirus for all the non-related samples, for which we used different analysis strategies along with MEGAHIT [33] and SPAdes [34].

## Results

### Assembly

Raw data obtained from eight human Norovirus samples passed FASTQC (v0.11.5, Babraham Bioinformatics) quality filters regarding the parameters per base sequence quality, per sequence average quality, N content and adapter sequences after the trimming steps described in the methods section. Mean read length was 100 bp as expected from library preparation. As shown in Table 1, sequencing experiments yielded a mean of 40 million total paired reads.

**Table 1:** General Assembly Statistics

	Sample A	Sample B	Sample C	Sample D	Sample E	Sample F	Sample G	Sample H	Average
Total number of reads	68.7 M	15.6 M	47.3 M	35.5 M	23.1 M	45.4 M	45.3 M	44.1 M	40.6 M
Ct value qRT-PCR	25.1	21.38	26.3	13.91	13.91	24.3	21.6	23.9	-
%Total NoV reads (paired, unique reads mapping norovirus)	18.5	15.6	1.4	93.9	34.4	42.9	98.6	76.5	47.7
%Total number of reads not mapping hs37d5	60.5	80.6	76	78.2	80	66.1	68.9	71.7	72.75
Completeness	YES	YES	YES	YES	YES	YES	YES	YES	-
Mean coverage depth against final contigs	453.05x	2690.55x	2587.67x	7671.9x	6309.99x	7712.23x	7805.97x	7743.57x	5371.87x
% of total reads identified by mapping (final contig)	0.026	15.79	1.43	94.31	34.32	42.97	98.77	76.93	45.57
Genotype	GII.17[P17]	GII.2[P2]	GII.17[P17]	GII.17[P17]	GII.17[P17]	GII.4[P4]	GII.4[P31]	GII.4[P4]	-
Final contig length (bp)	7551	7548	7560	7594	7589	7620	7674	7634	-
Published genomes assembly strategy+	pC SPAdes	pC MEGAHIT	pD SPAdes	pD MEGAHIT	pD MEGAHIT	pD MEGAHIT	pD MEGAHIT	pD MEGAHIT	-
% identity final contig against closest RefSeq reference*	99.6	98.6	99.6	99.5	98.5	98.1	98.5	98	-
number total variants above 1 % against final genome	230	180	187	15	55	43	23	32	95.6

NoV: norovirus, *closest reference norovirus genomes: -LC369255.1: samples A, C, D, E.; -MW305627.1: sample B ; -MW284782.1: samples F, H; -MW305617.1|: sample G. +(36,37) (note that these are not the best assembly strategies for each sample, more than one strategy yielded complete contigs)

**Fig. 1:**
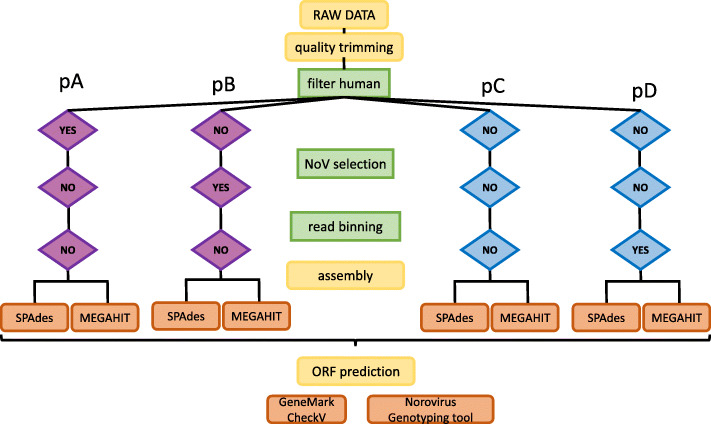
Analysis workflow. Yellow boxes are common steps and green boxes represent variable steps, indicated with YES or NO according to whether or not each step was performed in each approach. In all four workflows developed, final assembly was tested with both SPAdes and MEGAHIT

Different workflows with varying filtering steps before assembly were tested (Fig. 1). Identity percentages between the assembled genomes and the most related references from the viral RefSeq genomes database are represented in Table 1.

After the characterization with Norovirus genotyping tool of the longest NoV contig obtained by means of BLAST against the selected GenBank references (Additional File 1; filtered BLAST results for contigs >7 kb), completeness was assessed using checkV [35]. Four different strategies were tested: pipelines A, B, C or D (pA, pB, pC or pD, respectively). As complete NoV genomes were obtained from different strategies for the same samples (Table 2), final complete contigs were chosen and made publically available [36, 37]. The final contig chosen for sample A was from raw SPAdes, and CD-HIT SPAdes for sample C. For the rest, MEGAHIT was chosen: raw MEGAHIT in the case of sample B, and CD-HIT MEGAHIT in the case of samples D, E, F, G and H. (Table 1). Even though these were the final contig genomes published, all complete contigs are detailed in Table 2, and a summary with the average completeness per strategy can be found in Table 3. Identity percentages shared between final contigs and the rest of contigs yielded by different strategies are detailed in Additional File 2; alignment images between the main contig per strategy in a sample and the closest GenBank reference genome are presented in Additional files 8, 9, 10, 11, 12, 13, 14 and 15. In the case of SPAdes, only contigs obtained with custom kmer-lengths are indicated as autoadjusted kmer-lengths (21, 33, 55) used by default did not result in complete NoV genome contigs for most samples (Additional File 3).

**Table 2:** Summary of NoV assemblies in the approaches tested

	pA				pB				pC				pD			
	MEGAHIT		SPAdes		MEGAHIT		SPAdes		MEGAHIT		SPAdes		MEGAHIT		SPAdes	
	contig length	completeness	contig length	completeness	contig length	completeness	contig length	completeness	contig length	completeness	contig length	completeness	contig length	completeness	contig length	completeness
**A**	7549	100 %	^7550 K55	100 %	7621	100 %	^7548 K55-K77	100 %	7547	100 %	^7551 K55-K77	100 %	7163*	95.23	^7551 K55-K77	100 %
**B**	7823	100 %	7284*	96.52 %	7625	100 %	7549* K99	99.8 %	7548*	99.8 %	7705 K99	100 %	7588	100 %	^7506 K77*	99.26 %
**C**	7554	100 %	^7553 K77	100 %	7649	100 %	^7561 K77	100 %	7599	100 %	^7561 K77	100 %	7570*	97.8 %	^7560 K77	100 %
**D**	7728	100 %	7092*	93.96 %	7420*	98.6 %	7094*	93.99 %	7625	100 %	6898*	91.39 %	7594	100 %	7598 K77-K99	100 %
**E**	7596	100 %	7559 K99	100 %	7625	100 %	7502* K99	99.75 %	7594	100 %	7501* K99	99.74 %	7589	100 %	7556 K77	100 %
**F**	7684	100 %	6732*	89.31 %	7733	100 %	6732*	89.31 %	7705	100 %	6732*	89.31 %	7620	100 %	^7514 K77*	99.34 %
**G**	7457*	98.59 %	7587 K99	100 %	7227*	95.75 %	6216*	82.47 %	6871*	91.16 %	6222*	82.55 %	7674	100 %	7605 K77-K99	100 %
**H**	7714	100 %	7354*	97.23 %	7614	100 %	7338*	97.02 %	7518*	99.63 %	7338*	97.02 %	7634	100 %	7569 K99	100 %

* not complete. SPADes complete (or nearly complete) NoV genome contigs are shown with the kmer length that generated the contig. ^ contig lost in following kmer-length steps. pA, pB, pC and pD: pipelines A, B, C or D respectively. Genotypes are identical between pipelines, as indicated in Table 1

**Table 3:** Average completeness of NoV genomes in the approaches tested

	average completeness	
	MEGAHIT	SPAdes
pA	99.82 %	97.13 %
pB	99.30 %	95.30 %
pC	98.83 %	95 %
pD	99.14 %	99.83 %

. pA, pB, pC and pD: pipelines A, B, C or D respectively

Overall, MEGAHIT performed better than spades in most approaches, with at most 2 NoV contigs with length < 7.5 kb (1 in pA; 2 in pB; 1 in pC and 1 in pD). pA showed the best results for MEGAHIT with an average of NoV genome completeness of 99.82 %. 7/8 final NoV contigs were 100 % complete excepting sample G (98.59 %). In the case of SPADES, the strategies pA, pB and pC left 3 samples with genome completeness below 97 %. With the use of read binning (pD) along SPAdes 8/8 samples were completely assembled (samples B and F nearly complete in 99.26 and 99.34 % respectively).

Regarding pA, an average of 72.75 % of total reads were selected after removing host-mapped reads (detailed by sample in Table 1). These reads were selected for assembly using SPAdes and MEGAHIT.

With MEGAHIT, complete NoV contigs were obtained for 8/8 samples (sample G was nearly complete 98.59 %). In the case of SPAdes with custom kmer-lengths, genomes surpassing 7.5 kb were reached for 4/8 samples (A, C, E, G). Sample H had a level of completeness of 97.23 % (7354). The rest had a completeness of 96.52 % (B; length 7284), 93.96 % (D; 7092) and 89.31 % (F; 6732).

Percentages of unique reads mapping NoV reference genomes used in pB strategy were not even for the different samples as shown in Table 1. The average of these percentages was highly similar to that of the unique number of reads covering the complete genomes assembled (47 % vs. 45 %), indicating that NoV reads mapped successfully against the references used and that is resembled in the completeness achieved with this strategy. Adjusted kmer-lengths for SPAdes accomplished contigs over 7.5 kb in 4/8 samples NoV genomes (A 100 %, B 99.8 %, C 100 % and E 99.75 %). The rest were sample H (covered 97.02 %; length 7338), D (covered 93.99 %; length 7094), F (covered 89.31 %; length 6732) and G (covered 82.47 %; length 6216). In the case of MEGAHIT, 6/8 complete genomes were reached. The rest, sample D and G had a completeness of 98.6 (length 7420) and 95.75 % (length 7227), respectively.

pC consisted of the assembly of trimmed raw reads with MEGAHIT and SPAdes without additional read filtering. In the case of MEGAHIT 7/8 samples were completely assembled. Sample G NoV genome was incomplete with length 6781 (completeness 91.16 %). Two others had levels of completeness below 100 (sample B 99.8 %; sample H 99.63 %). SPAdes accomplished 7.5 kb genomes in 4/8. From the incomplete genome samples, H had a level of completeness of 97.02 % (contig length 7338), whereas D, F, G were below 7 kb and completeness percentages were 91.39, 89.31 and 82.55, respectively.

Regarding pD, after read-binning, MEGAHIT assemblies yielded 7/8 NoV genomes over 7.5 kb. The incomplete sample was sample A with genome length 7163 (95.23 % complete). Sample C was nearly covered at 97.8 %; length 7570. In the SPAdes counterpart 8/8 samples were completely assembled (sample B 99.26 % and sample F 99.34 % nearly complete).

### Assembly statistics

We compared assembly qualities between MEGAHIT and SPAdes data from the approaches tested. Tables 4 and 5 show the results obtained. Tables with assembly statistics per sample are in Supplementary Data (Additional File 4 for all obtained contigs and Additional File 5 for Norovirus contigs).

**Table 4:** General statistics from all contigs retrieved in the assembly approaches

	Mean total number of contigs	Mean number of contigs >5 kb	Mean number of contigs >10Kb	Mean total length of contigs > 5 kb	Mean total length of contigs > 10 kb	Mean largest contig	Mean N50 value length
Human filter MEGAHIT (pA)	6851	16	1	114,901	25,288	20,422	1057
NoV filter MEGAHIT (pB)	734	3	0	18,951	0	7753	1257
Raw MEGAHIT (pC)	8142	18	1	132,123	28,435	20,662	1001
MEGAHIT + CD-HIT (pD)	6402	15	1	115,282	27,543	19,538	1018
Human filter SPAdes (pA)	11,250	3	0	25,351	6508	10,274	890
NoV filter SPAdes (pB)	1404	1	0	7335	0	6681	1146
Raw SPAdes (pC)	13,175	4	1	31,236	9095	9558	852
SPAdes + CD-HIT (pD)	11,948	13	1	89,159	13,571	13,530	928

pipelines A, B, C or D respectively

**Table 5:** General statistics from all contigs assigned to Norovirus using BLAST algorithm

	Mean total number of contigs	Mean number of contigs >5 kb	Mean number of contigs >10Kb	Mean total length of contigs > 5 kb	Mean total length of contigs > 10 kb	Mean largest contig	Mean N50 value length
Human filter MEGAHIT (pA)	27	1	0	9282	0	7638	4567
NoV filter MEGAHIT (pB)	14	1	0	9527	0	7286	4953
Raw MEGAHIT (pC)	18	2	0	10,153	0	7501	4555
MEGAHIT + CD-HIT (pD)	34	1	0	10,700	1635	8293	5010
Human filter SPAdes (pA)	138	1	0	6099	0	6556	3632
NoV filter SPAdes (pB)	131	1	0	7335	0	6681	3861
Raw SPAdes (pC)	138	1	0	7954	0	6623	3733
SPAdes + CD-HIT (pD)	16	1	0	7273	0	6332	3682

. pA, pB, pC and pD: pipelines A, B, C or D respectively

Regarding MEGAHIT, the total number of contigs obtained in pD was reduced by 22 % compared to pC. From the total number of assembled contigs, 0.22 % belonged to NoV in pC, whereas using CD-HIT (pD) this value was 2.41 times higher (0.53 %), being the number of NoV contigs 1.88-fold higher in pD. Mean N50 was also increased with CD-HIT to 5010 in NoV contigs (pC mean N50 4554), improving the assembly from 98.83 to 99.14 % average completeness.

MEGAHIT assembled Norovirus whole genomes in the whole set of samples with pA (sample G 98.59 % completed). Average completeness for this approach was the highest for MEGAHIT (99.82 %). Average NoV contigs N50 value was 4567, highly similar to that in pC, and average NoV contigs proportion (0.39) was lower to that in pD. The number of contigs respecting to pC was reduced by 16 %. pB MEGAHIT had an average genome completeness of 99.30 %. The number of total contigs was 9-fold lower than pC and from these, 1.8 % belonged to NoV.

MEGAHIT performed successfully assembling complete NoV genomes along all the approaches and even though pC had the lowest average completeness, the results are almost equivalent in the 4 strategies.

Regarding SPAdes, the percentage of NoV contigs was 7.7-fold higher in pC (1 %) than pD (0.13 %). The total number of NoV contigs was reduced by 11 % using pD. However, the proportion of useful NoV contigs (>5000pb) was 7.75 times higher in pD. N50 was similar in the two approaches considering all contigs and only norovirus-matching contigs. With pC, (average completeness 95 %), 4/8 samples had contigs longer than 7000 bp (3 completely assembled and 1 nearly assembled 99.74 % sample E), whereas with pD the average completeness was 99.83 % and 8/8 samples had a Norovirus whole-genome candidate contig, surpassing 7.5 kb length. 6/8 of them accomplishing complete NoV genomes and 2/8 nearly assembled (B 99.26 %; F 99.34 %). In this case strategies pA and pB did not present any advantage compared to pC which is the simplest approach. The level of completeness in pB was 95.3 % and even though in pA this value was improved (97.13 %), 4/8 samples do not reach NoV contig lengths over 7.5 kb (so as in pC and pB). The proportions of NoV contigs over 5 kb were 0.6 and 0.8 % in pA and pB for SPAdes. Again, this proportion was 11 and 8-fold higher in pD (pipelines A, B and C produced more NoV contigs but only a few reached completeness, whereas in pD the number of total NoV contigs was lower).

### SPAdes kmer-lengths

Spades autoadjusted kmer-lengths used for assembly regarding read length (100 bp) to 21, 33 and 55 were used for the assembly at first but SPAdes did not yield contigs near 7Kb in the majority of samples (Additional file 3). After obtaining notable differences in the number of assembled NoV genomes using SPAdes versus MEGAHIT we sought to test whether these differences were due to the kmer-lengths used by each assembler. Kmer-lengths used by MEGAHIT were 21, 29, 39, 59, 79, 99 and 119. For that reason, we tested all the strategies with SPAdes using a set of kmer-lengths of 21, 33, 55, 77, 99 and 119 (intermediate kmer-lengths chosen are the recommended in the SPAdes manual for 250 × 2 bp read lengths). The last kmer-length was not used as it surpassed read length. The rest of the parameters used were the same and the option –meta was also included. As shown in Table 2, contigs over 7k were accomplished but excepting pD strategy, only half of the samples NoV genomes were assembled. In other words, complete assemblies were only reached with pD strategy and custom kmer-lengths. Besides, some assemblies were reached at different kmer-lengths and in certain cases, these were lost in the following kmer-length steps (Table 2 shows kmer-lengths at which the contig was assembled in the case of SPAdes complete or nearly complete contigs; contigs marked with ^ are lost in the following kmer steps), not reporting the longest contig in the final assembly FASTA file. In the case of MEGAHIT, all contigs generated are reported in the final assembly FASTA file. Despite the fact that pD was the strategy with more assembled NoV genomes, 4/8 samples are assembled in an intermediate kmer-length that was no longer reported in subsequent assembly steps.

Sample A was completely assembled with SPAdes pC with the use of default kmers (additional File 3). As it can be seen in Table 2 is the only sample appearing complete at step K55, which was the last used by default SPAdes.

### Variants

**Fig. 2:**
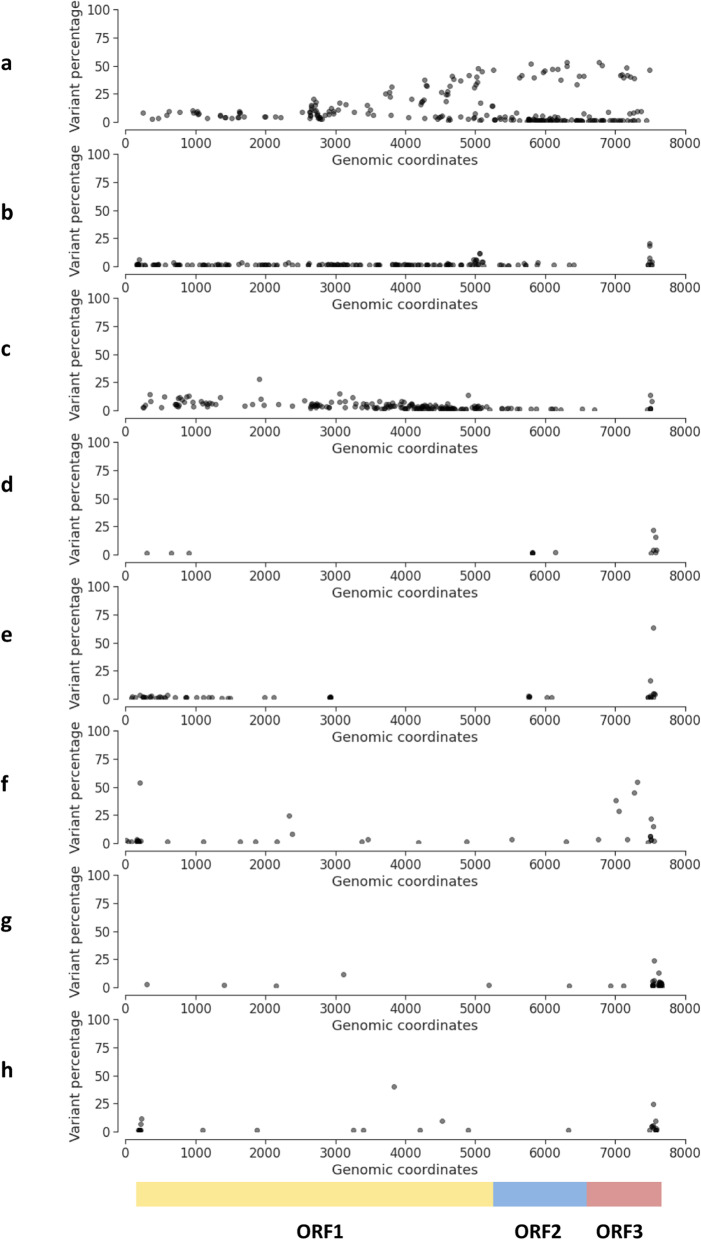
Variant frequencies across assembled Norovirus genomes on each individual (**A** to **H**)

The eight studied samples had an average number of 95.6 mutations against the final assembled genomes (including variants present in over 1 % of reads). The number of variants found against the final contigs for each sample were 230, 180, 187, 15, 55, 43, 23, 32 in the order A-H. A, B and C exhibited the highest number of variants (230, 180 and 187) over 1 %. However, when considering higher variant frequencies (>=10 %), only sample A maintains high variability, being the number of nucleotide changes identified 70, 5, 13, 3, 1, 7, 4, 3 variants in order from A to H. In general, variation frequencies are higher towards the 3’ end of the virus genome (Fig. 2).

Sample A could exhibit a co-infection of various NoV strains due to the presence of a high number of nucleotide changes with respect to the final contig assembled (variant frequencies ranging from 10 to 57 %; Fig. 2). Interestingly, all the strategies shown in Table 2 result in the assembly of the same contig genotype although they represent a small part of the reads corresponding to NoV strains. The percentage of total reads covering the final contig with genotype GII.17 was 0.026 %. In Additional File 6 the 20 GenBank NoV references with most reads mapped for all samples are reported, showing different genotypes in the case of sample A (GII.12, GII.4, GII.17, GII.3, GII.2), whereas in the rest the majority were consistent with the final contig genotype.

### Computation resources

We compared the computational resources used by both MEGAHIT and SPADES with the raw assembly and after read-binning. MEGAHIT in pC used maximum 50Gb memory for the four samples tested, taking 7 h to complete assembly (1.75 h/sample). Use of the 16 CPUs was 12 %. With the pD strategy, it required 20 % CPU and a peak memory of 60Gb, assembling all tested samples in just 1 h.

We studied the time and resources used by SPAdes with four test samples using intra-sample threading. It took 8 days to assemble these samples (mean, 44.25 h per sample) without error correction, 186 Gb of memory and 16 threads with metaSPAdes. Use of CPU was 100 % with peak memory of 160Gb. With the pD binning strategy, the same test required 30 min for all samples with 45Gb peak memory and maximum CPU usage under 10 %.

## Discussion

Different strategies are needed for de novo genome assembly, especially for RNA virus genomes, which present a higher substitution rate than any other microorganism (10^−3^-10^−5^/site/year) [22, 23, 38–40]. Although there are plenty of assemblers and even specific viral assembly pipelines [41, 42], they do not always ensure genome completion. Due to the variable nature of metagenomic data, there is no strict workflow to obtain the best assembly and results show high variability. Even with samples treated equally, different assembly strategies may be required to obtain optimal results. Data exploration prior to data analysis is crucial and rigorous analysis is needed to achieve genome completion. Accordingly, in this study we explored several different strategies used to assemble Norovirus genomes in non-related patients.

Interspecies and intraspecies variability was a major limitation in the analysis, and the diversity present in our data is further confirmed by the low proportion of contigs belonging to NoV in Table 1. Assembly quality did not present any advantage with respect to the raw assembly pipeline when a specific NoV reference-oriented analysis was performed. Only the host-reads filter presented improvements when filtering or gathering reads that could belong to Norovirus in the case of MEGAHIT. When attempting to select reads mapping against NoV genomes, a great number of reads were filtered out, with a highly variable percentage of mapped reads between samples (Table 1). Neither were advantages found when removing human reads (pA) in the case of SPAdes, even though some studies have used mapping to host genomes to remove non-viral reads. Several studies address the use of reference-guided assemblies to reconstruct viral genomes. However, the presence of intra-host variability can cause biased alignments and references have to be chosen carefully [43, 44].

The mRNA isolation strategy enabled Norovirus viral representation with read fractions ranging from 0.02 to 98.5 % due to a large variability in virus load of each patient (Table 1, total Norovirus reads covering contig and Ct value qRT-PCR). The variability in Norovirus representation suggested a need for metagenomic assembly, for which purpose we preferred to use the “meta” varieties in the case of SPADEs, expecting that contigs and scaffolds for other organisms would also be present.

Our strategy to counteract this variability was to use a clustering algorithm to reduce raw data complexity, selecting for the purpose CD-HIT, a tool widely used in metagenomics specifically for reducing redundancy and sequencing replicates in metagenomic samples.

Among assemblers, MEGAHIT performed better than SPAdes, as samples were successfully assembled independently of CD-HIT use (all strategies were successful). CD-HIT was most advantageous when applied before SPAdes assembly, as it is computationally more expensive and could not yield Norovirus contigs reaching 7.5 kb in 4 samples without read binning in any of the approaches (Table 2). By default, SPAdes worked better with samples with lower coverage (Table 1; the only sample (A) fully assembled with raw SPAdes with the default autoadjusted kmer-lengths up to 55 had 455x mean coverage, 11-fold lower than the average coverage obtained in all samples). Samples with higher coverage obtained better assembly results after kmer-length 77 and read binning. The stringency used along CD-HIT may vary depending on the raw data and tuning steps are advisable to avoid the loss of coinfecting strains.

As reported in previous studies [14, 32, 45], SPAdes is a widely-used short-read assembler for general use in viruses, including Norovirus studies. Nevertheless, in our specific scenario we obtained more optimal results with MEGAHIT. All approaches assembled completely 6/8 samples with MEGAHIT. Moreover, pC MEGAHIT assemblies improved slightly when combined with CD-HIT (pD) in completeness, and also regarding the previously described efficient performance and low computational resource requirements. Our data thus support use of MEGAHIT for in-depth Norovirus assembly and by extension for other RNA viruses with high sequencing depths, whereas SPAdes will perform optimally with lower-coverage sequencing experiments. Combined with a step to reduce sequence redundancy, SPAdes will improve assembly quality while reducing data complexity [26, 46, 47].

## Conclusions

We tested different workflows for the accurate and complete assembly of Norovirus genomes. These included different filtering steps and their subsequent assembly with both SPAdes and MEGAHIT. Even though there is no universal workflow for viral RNA assembly, NoV genome-oriented strategies did not present advantages compared to assembly without filters for any of the strategies, with the exception of host-filtering for MEGAHIT. We describe the performances of MEGAHIT and SPAdes and the use of read-binning to improve assembly statistics.

## Methods

### Norovirus-targeted Next Generation Sequencing (NGS)

Fecal samples from eight patients affected by acute non-bacterial gastroenteritis were collected for Norovirus study and metagenomic analysis from November 2015 to September 2017. All patients were treated in Hospital Clínico Universitario of Valencia, Spain. The present study was carried out in accordance with the Declaration of Helsinki and was approved by the Ethics Committee of the Hospital Clínico Universitario of Valencia (Approval No. F-CE-GEva-15). Patients accepted to participate and gave their written consent.

Samples were processed using Trizol (Invitrogen Corp.) for RNA extraction according to the manufacturer’s instructions. Sequencing libraries were prepared for Norovirus sequencing by means of polyA enrichment (TruSeq RNA Sample Prep Kit v2, Illumina, California, EEUU) and sequenced by Macrogen (Seul, South Korea). Samples were sequenced on an Illumina HiSeq 2000, obtaining paired end reads with an average length of 100 bp. Raw data from all samples is available at the sequence read archive (SRA), accession number PRJNA497363. GenBank Accessions: sample A: MH997861, sample B: MK789430, sample C: MK789431, sample D: MK789432, sample E: MK789433, sample F: MK789434, sample G: MK789435, and sample H: MK789436 (the strategies chosen for the final published genomes are detailed in Table 1).

### Norovirus genome assembly

All assembly steps were performed on a local server (16 Intel ® Xeon ® CPU E5-2650 0 @ 2.00 GHz processors, 190 GB of RAM and 41 TB disk space) using 16 CPU threads. Use of computational resources was coordinated using GNU Parallel [48]. Tests with a fraction of the samples were performed to study time and RAM memory required to complete assemblies using NMON v14g [49] with both assembly from raw reads (pC) and read binning (pD).

Before assembling the Norovirus genomes, we performed read quality control using FASTQC (v0.11.5, Babraham Bioinformatics), and quality filtering using seqtk 1.2-r101-dirty with the default parameters (trimming up to 30 bp from each side following a 0.05 error rate threshold) [50].

Metagenome assemblies were performed on quality-trimmed FASTQ files using metaSPAdes v3.11.1 [51] with auto adjusted k-mer lengths of 21, 33 and 55 nucleotides and in parallel, using MEGAHIT v1.1.3 [33] with optimized k-mer lengths of 21, 29, 39, 59, 79, 99 and 119. After incompleteness of SPAdes NoV contigs, we decided to test kmer-lengths 21, 33, 55, 77, 99 and 119. The longest kmer was chosen to be equal to MEGAHIT and the rest are the recommended in SPAdes manual for Illumina longer reads (250 × 2).

Four variations of the assembly strategy were implemented to obtain Norovirus genomes (Fig. 1). Since our biological data is poly-A RNA, we expected to find human mRNA representation. Therefore, in the first derivation (pipeline pA), human reads were removed (Fig. 1; pA). Trimmed FASTQ files were mapped on the hs37d5 reference assembly (ftp://ftp.1000genomes.ebi.ac.uk/vol1/ftp/.../hs37d5.fa.gz) using BWA *mem* v0.7.25-r1140 [52]. Reads not mapped against this reference were selected for assembly using both MEGAHIT and SPAdes with the previously described parameters, as well as --meta flag in SPAdes.

Pipeline pB directly selected Norovirus reads from the original FASTQ files by mapping against GenBank NoV genome sequences (483 genomes: updated in July 2021; Additional file 7 includes accession numbers) (Fig. 1; pB). Trimmed FASTQ files were mapped against the former FASTA reference file as in pA, selecting mapped reads against the NoV genomes reference for assembly in this case. Pipeline pC consisted of assembling trimmed FASTQs without applying any filtering steps (Fig. 1; pC).

Finally, pipeline pD consisted of performing sequence binning via CD-HIT [53] on the raw quality-trimmed FASTQ files, clustering sequencing reads at 80 % identity to reduce sequence redundancy. SPAdes and MEGAHIT were run with the same parameters as previously used for a final round of metagenome assembly, using CD-HIT preprocessed FASTQ files as input (Fig. 1; pD).

Norovirus contigs were identified using a local BLAST database built from reference Norovirus genomes obtained from GenBank (483 genomes: updated in July 2021; disclosed in Additional File 7). All assembled contigs were subjected to BLASTN [54] v2.2.31+ search, and Norovirus contigs were retrieved.

Quality statistics from all generated assemblies and filtered Norovirus contigs were assessed using QUAST v4.6.3 [55]. Raw quality-trimmed sequencing reads were mapped to the assembled Norovirus contigs using BWA *mem* to assess the volume of reads corresponding to Norovirus.

Mean depth of coverage for each complete Norovirus genome per sample was calculated using Samtools v1.7 [56], on BAM files generated by mapping all sample reads to their corresponding final de novo assembled genomes using BWA *mem*. Open reading frames were predicted with GeneMark v3.25 [57] and inspected using Norovirus Genotyping tool v2.0 [58]. Final assemblies were chosen according to comparisons based on N50, contig length and completeness assessed with checkV [35].

For the sake of comparing the accuracy of the two assemblers used, the different workflows tested were compared (Fig. 1).

### Norovirus variability among samples

After raw read mapping to the final assembled Norovirus contigs, variant calling was performed using Freebayes v1.2.0 [59]. Only variants at 1 % VAF and at least six alternate reads were considered.

## Supplementary information

**Additional file 1.** BLAST results for contigs >7 kb obtained with the 4 approaches and 2 assemblers tested.

**Additional file 2.** Identity percentages shared after local alignment between the published genomes per sample and the rest of main contigs yielded per strategy.

**Additional file 3.** Longest Norovirus contigs retrieved from all strategies with default kmer-lengths used by SPAdes.

**Additional file 4.** Results per sample in all contigs.

**Additional file 5.** Results per sample in Norovirus contigs.

**Additional file 6.** 20 most mapped Norovirus GenBank references per sample.

**Additional file 7.** GenBank accessions of the reference NoV genomes employed.

**Additional file 8.** Contig alignments of sample A, against the closest reference from GenBank.

**Additional file 9.** Contig alignments of sample B against the closest reference from GenBank.

**Additional file 10.** Contig alignments of sample C against the closest reference from GenBank.

**Additional file 11.** Contig alignments of sample  D against the closest reference from GenBank.

**Additional file 12.** Contig alignments of sample E against the closest reference from GenBank.

**Additional file 13.** Contig alignments of sample F against the closest reference from GenBank.

**Additional file 14.** Contig alignments of sample G against the closest reference from GenBank.

**Additional file 15.** Contig alignments of sample H against the closest reference from GenBank.

## Data Availability

The datasets supporting the conclusions of this article are available at GenBank [60]. The GenBank accession numbers for the norovirus genomes are MH997861, MK789430, MK789431, MK789432, MK789433, MK789434, MK789435, and MK789436. The sequence data are available in the Sequence Read Archive (SRA) under BioProject number PRJNA497363.

## Abbreviations

HTS: High-Throughput Sequences
ORF: Open Reading Frame
pA, pB, pC or pD: pipelines A, B, C or D, respectively
SNVs: Single Nucleotide Variants
VAF: Variant Allele Frequency
