# Supplementary material for: Benchmarking different approaches for Norovirus genome assembly in metagenome samples
Source: BMC Genomics. 2021 Nov 24;22:849. doi: 10.1186/s12864-021-08067-2 (PMC8611953; doi:10.1186/s12864-021-08067-2)

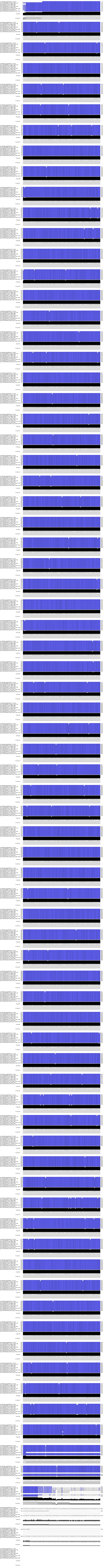

Supplement: Supplementary file 9 — Additional file 9. Contig alignments of sample B against the closest reference from GenBank. [file 12864_2021_8067_MOESM9_ESM.pdf]
