# Supplementary material for: Benchmarking different approaches for Norovirus genome assembly in metagenome samples
Source: BMC Genomics. 2021 Nov 24;22:849. doi: 10.1186/s12864-021-08067-2 (PMC8611953; doi:10.1186/s12864-021-08067-2)
Supplement: Supplementary file 11 — Additional file 11. Contig alignments of sample D against the closest reference from GenBank. [file 12864_2021_8067_MOESM11_ESM.pdf]

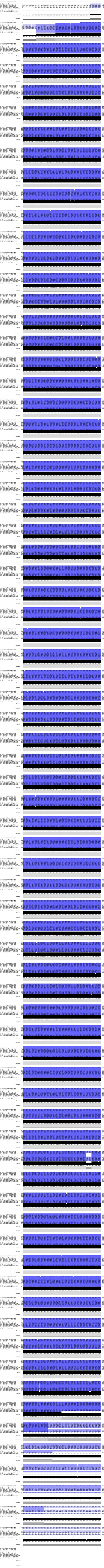
gi|1761024121|dbj|LC369255.1|/1-7588
D_pD_MEGAHITk119_len=7594/1-7594
D_pB_MEGAHITk119_len=7420/1-7420
D_pA_MEGAHITk119_len=7728/1-7728
D_pC_MEGAHITk119_len=7625/1-7625
D_pA_SPADESNODE_2_length_7092/1-7092
D_pD_SPADESNODE_18141_length_7598/1-7598
D_pB_SPADESNODE_1_length_7094/1-7094
D_pC_SPADESNODE_3_length_6898/1-6898
Consensus
Occupancy
